# Synergistic Therapeutic Effects of Tetrahydroberberine Combined with Protopanaxadiol on PCPA-Induced Insomnia in Rats: Involvement of the Microbiota–Gut–Brain Axis and Regulation of PI3K/AKT/AGE-RAGE Pathways

**DOI:** 10.3390/ph19030390

**Published:** 2026-02-28

**Authors:** Meijia Li, Ying Wang, Zixia Liang, Honghua Li, Yun Zhao, Ling Kong, Na Guo, Guoxin Dai, Guimin Zhang, Xiaoyan Lu, Jingchun Yao

**Affiliations:** 1 College of Chinese Pharmacy, Tianjin University of Traditional Chinese Medicine, Tianjin 301600, China; 17332326437@163.com; 2 School of Medicine and Pharmacy, Ocean University of China, Qingdao 266003, China; 3 Chinese Pharmacy, Guangdong Pharmaceutical University, Guangzhou 510006, China; 4 State Key Laboratory of Integration and Innovation of Classic Formula and Modern Chinese Medicine, Lunan Pharmaceutical Group Co., Ltd., No. 209 Hongqi Road, Linyi 276000, China; 5 School of Pharmacy, Heilongjiang University of Chinese Medicine, Harbin 154600, China; 6 Lunan Better Pharmaceutical Co., Ltd., No. 209 Hongqi Road, Linyi 276000, China

**Keywords:** insomnia, tetrahydroberberine (THB), protopanaxadiol (PPD), microbiota–gut–brain axis (MGBA), PI3K/AKT pathway, rat model

## Abstract

**Aim:** This study investigated the synergistic therapeutic effects and underlying mechanisms of tetrahydroberberine (THB) combined with protopanaxadiol (PPD) on p-chlorophenylalanine (PCPA)-induced insomnia in rats. **Methods:** Rats were randomly divided into normal, model, diazepam, THB monotherapy, PPD monotherapy, and THB + PPD combination groups. Evaluations included the pentobarbital sleep test, HE staining, ELISA, 16S rRNA sequencing, metabolomics, and Western blot. **Results:** Results demonstrated that the THB + PPD combination exhibited significant synergistic effects compared with monotherapies: the combination shortened sleep latency by 56.2% (vs. 44.2% for THB alone and 20.7% for PPD alone) and prolonged sleep duration by 112.8% (vs. 70.2% for THB and 59.6% for PPD) relative to the model group, while effectively restoring body weight gain. Histologically, combined treatment significantly alleviated hippocampal neuronal damage and increased the number of intact neurons in the dentate gyrus. Molecularly, it upregulated brain-derived neurotrophic factor (BDNF) and glial cell line-derived neurotrophic factor (GDNF) levels, restored neurotransmitter balance (serotonin, dopamine, and glutamate), suppressed overactivation of the hypothalamic–pituitary–adrenal (HPA) axis (reducing corticotropin-releasing hormone and corticosterone), and decreased pro-inflammatory cytokine expression. Gut microbiota analysis revealed that the combination restored microbial homeostasis (increasing beneficial bacteria such as *Lactobacillus*) and modulated the glycine–serine–threonine metabolic pathway. Mechanistically, THB + PPD synergistically activated the PI3K/AKT neurotrophic pathway (p-PI3K and p-AKT expression increased by 1.9-fold and 2.5-fold, respectively, vs. model), inhibited the AGE/RAGE pro-inflammatory axis (RAGE expression decreased by 31.8%), and enhanced blood–brain barrier integrity by upregulating tight junction proteins (ZO-1, Occludin). **Conclusions:** THB combined with PPD exerts synergistic anti-insomnia effects through multi-level regulation of the microbiota–gut–brain axis, neurochemical balance, and key signaling pathways, providing a promising foundation for developing safe natural product-based combination therapies.

## 1. Introduction

Insomnia is a highly prevalent global public health issue that has become a critical challenge affecting population health and quality of life. Approximately 33% of the global general population experiences at least one insomnia symptom, and 8–18% of individuals report dissatisfaction with subjective sleep quality. This proportion is even higher in groups such as the elderly and patients with chronic diseases [1]. Insomnia patients often present with daytime fatigue, impaired cognitive functions (attention and memory), emotional lability or depression, and some also suffer from reduced work efficiency and impaired social functioning [2]. Beyond these clinical manifestations, long-term sleep disturbance triggers biological changes including elevated serum inflammatory factors and decreased immune cell activity, and significantly increases the risk of comorbidities such as depression [3] (2–3 times higher than the general population), hypertension, coronary heart disease, arrhythmia, and heart failure. In contrast, normal sleep rhythm is crucial for maintaining physiological functions—it promotes cardiovascular homeostasis and cognitive memory consolidation, regulates immune cell activity to reduce susceptibility to infectious diseases, and mitigates the risk of developing major diseases such as cancer and depression. Thus, improving insomnia is of great significance for safeguarding overall health.

The pathogenesis of insomnia involves the interaction of multiple factors, including genetic susceptibility, environmental stress, behavioral habits, and physiological homeostasis imbalance [4]. Its core pathological feature is the hyperarousal state of the central nervous system. Current research has identified several key pathways closely associated with insomnia [5]: hyperactivation of the hypothalamic–pituitary–adrenal (HPA) axis can enhance central arousal signals, directly inducing or exacerbating insomnia; abnormal function of the type A receptor of γ-aminobutyric acid (GABA), the main inhibitory neurotransmitter in the central nervous system, weakens central inhibitory effects and is directly linked to the development of neuropsychiatric diseases such as insomnia and anxiety; and the 5-hydroxytryptamine (5-HT) system participates in the pathological process of insomnia by regulating the wake–sleep cycle—while its classical role is to promote wakefulness and inhibit rapid eye movement sleep, activation of specific subtype receptors (e.g., 5-HT1A) can also exert sleep-promoting effects, reflecting the complexity of its function.

Molecules involved in innate immunity exhibit a bidirectional interaction with sleep and circadian oscillators, and inflammatory markers represented by IL-1β, TNF-α, and IL-6 play a central role in the pathological progression of insomnia. Changes in the levels of these pro-inflammatory factors can directly reflect the severity of insomnia and the status of neuroinflammation: increased waking activity or pathogen stimulation can activate their expression, while the inflammatory regulatory disorders induced by insomnia in turn exacerbate sleep disturbances, forming a vicious cycle. Additionally, inflammatory dysregulation serves as a key link between insomnia and physical diseases—peripheral and central inflammatory factors [6] (e.g., IL-1β and TNF-α) can directly regulate sleep structure by affecting cerebral blood flow and altering electroencephalogram power spectra, providing an explanation for the comorbidity mechanism between insomnia, cardiovascular diseases, and depression. It is thus evident that reducing pro-inflammatory factor levels is one of the key molecular indicators for evaluating the efficacy of anti-insomnia therapies, and targeted regulation of the expression and function of inflammatory markers is expected to become a potential therapeutic strategy for improving insomnia and alleviating its comorbidities [7].

The gut harbors approximately 100 trillion microbes, and the discovery of their impact on health and disease has spurred a boom in multidisciplinary research. A close bidirectional regulatory pathway exists between the gut and the central nervous system (CNS): a balanced gut microbiota is essential for normal brain physiology and emotional functions, while the CNS also regulates most gastrointestinal physiology. Disruption of this pathway can induce neurological and gastrointestinal diseases in both directions.

In recent years, advances in research on the microbiota–gut–brain axis (MGBA) have provided a new perspective for insomnia intervention [8]. This axis enables bidirectional communication between the gut microbiota and the central nervous system through three major pathways: neural (e.g., vagus nerve), immune (e.g., inflammatory factors), and metabolic (e.g., short-chain fatty acids and neurotransmitter precursors), playing a core role in neural development, emotional regulation, and cognitive function maintenance. Animal experiments have confirmed that gut microbiota depletion (e.g., induced by antibiotics) can significantly disrupt normal sleep rhythms, leading to sleep fragmentation and reduced total sleep time [9]. This suggests that gut microbiota homeostasis is an important basis for maintaining normal sleep and provides a theoretical foundation for developing insomnia intervention strategies from the MGBA perspective.

Key mediators of each pathway regulate sleep–wake rhythms through the MGBA: the vagus nerve transmits gut microbiota signals to sleep-regulating centers in the CNS; pro-inflammatory factors interfere with central neuroinflammatory status via the immune pathway; and metabolites such as short-chain fatty acids (SCFAs) and tryptophan derivatives modulate melatonin secretion and neurotransmitter balance. Animals with insomnia models often exhibit gut microbiota imbalance (abnormal Bacteroidetes/Firmicutes ratio), while probiotic supplementation can restore sleep structure. This confirms that MGBA dysfunction is a crucial pathological mechanism of insomnia, offering a new direction for targeted intervention [10].

In clinical treatment, Cognitive Behavioral Therapy for Insomnia (CBT-I) is recommended as a first-line treatment by guidelines worldwide due to its stable long-term efficacy and minimal side effects [11]. However, CBT-I has significant limitations in practical application: its treatment course lasts 4–8 weeks and requires operation by professional physicians, leading to high treatment costs and poor accessibility in primary healthcare settings; moreover, existing studies mostly use young and healthy individuals as subjects, resulting in insufficient efficacy data for elderly insomnia patients or those with comorbid physical diseases, and limited sample representativeness [12]. Therefore, pharmacotherapy remains a widely used intervention in clinical practice due to its advantages of rapid onset and ease of use. Currently, commonly used clinical drugs for insomnia include benzodiazepines (e.g., diazepam), melatonin receptor agonists (e.g., daridorexant), orexin receptor antagonists (e.g., suvorexant), and antidepressants (e.g., trazodone) [13]. However, these drugs generally have clinical drawbacks: short-term use may improve sleep parameters, but long-term application is prone to reduced tolerance, efficacy attenuation, and side effects such as cognitive impairment, rebound insomnia, fall risk (especially in the elderly), and drug dependence. Furthermore, no global clinical consensus on the “optimal efficacy-risk ratio” for insomnia medications has been reached, which further limits their rational use. Thus, developing safe, effective and novel therapeutic strategies is of great clinical significance for insomnia treatment [14].

Based on the above research background, this study selected protopanaxadiol (PPD) and tetrahydroberberine (THB) as combined intervention drugs. PPD is the main active metabolite of ginsenosides in vivo, which is obtained by acid or base hydrolysis of ginsenosides (the core active components of Panax genus plants such as Panax ginseng and Panax notoginseng). Compared with ginsenosides, PPD has improved bioavailability due to its enhanced membrane permeability and resistance to gastrointestinal tract interference, and its stable skeleton and reactive sites (e.g., active hydroxyl group at C-3 position) also support structural modification for better activity [15]. Previous studies have confirmed that PPD exerts antidepressant effects by inhibiting central inflammatory responses and upregulating the expression of neurotrophic factors (e.g., BDNF); its anti-inflammatory and neuroprotective properties are precisely the core targets for insomnia treatment. Meanwhile, preclinical studies suggest that ginsenoside components can improve sleep structure in animal models, further supporting PPD’s potential for insomnia intervention [16]. In this review, the pharmacological activities of panaxadiol, protopanaxadiol and their structurally modified derivatives are comprehensively summarized.

As a hydrogenated derivative of berberine, THB has well-documented central inhibitory effects, which can target the core pathological feature of insomnia—central hyperarousal [17]: animal experiments have shown that after intravenous or oral administration, THB can significantly inhibit the electrical activity of the motor cortex and hippocampus in rabbits and rats, reducing central excitability. This is highly consistent with the “central hyperarousal” pathological feature of insomnia and implies a potential mechanism for improving sleep.

Against this backdrop, we proposed a multi-target synergistic strategy for insomnia treatment based on the distinct pharmacological properties of PPD and THB. Based on the distinct pharmacological properties of protopanaxadiol (PPD) and tetrahydroberberine (THB), we proposed a multi-target synergistic strategy for insomnia treatment. PPD focuses on anti-inflammatory regulation and neurotrophy by inhibiting central inflammation and upregulating BDNF expression to target insomnia’s neuroinflammatory core and repair damaged neurons, while THB emphasizes central inhibition and neuroprotection by regulating neurotransmitter balance (e.g., γ-aminobutyric acid) to act directly on the sleep–wake center. Their combination achieves synergistic complementarity, comprehensively covering insomnia’s multi-link pathological mechanisms—simultaneously downregulating HPA axis hyperactivity, suppressing neuroinflammation, enhancing central inhibition, and protecting neurons—to break through monotherapy limitations and improve efficacy.

Although the single-drug pharmacological effects of PPD and THB have been preliminarily reported, the synergistic effect of their combined application in insomnia treatment and whether they exert effects by regulating the microbiota–gut–brain axis (MGBA) have not been reported to date. In this study, PPD combined with THB was used as the intervention, and a rat model of insomnia was established by intraperitoneal injection of p-chlorophenylalanine (PCPA). Integrating behavioral tests, molecular biology techniques, pathological detection, and multi-omics techniques, we systematically investigated the synergistic therapeutic effects and underlying mechanisms of the combined administration from multiple dimensions, including sleep behavior, neuroinflammation, gut microbiota-metabolite homeostasis, and central signaling pathways. This study aims to provide a new drug combination strategy and theoretical basis for the clinical treatment of insomnia.

## 2. Results

### 2.1. Combined Treatment with THB and PPD Improves the Behavioral and Physiological Phenotypes of Insomniac Rats

**Figure 1:**
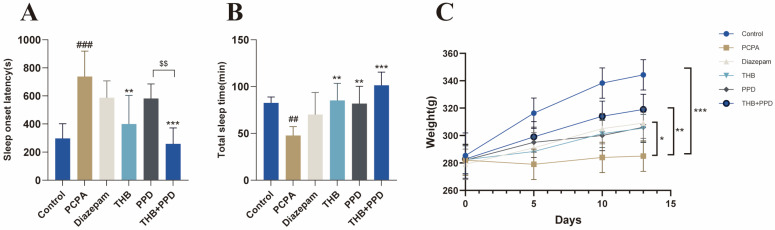
THB combined with PPD improves sleep behavior and overall physiological state of insomniac rats: (**A**) Sleep latency (SL) of rats in each group (*n* = 6). (**B**) Sleep duration (SD) of rats in each group (*n* = 6). (**C**) Body weight changes of rats in each group during the experimental period (*n* = 6). Data were analyzed by one-way analysis of variance (ANOVA) followed by Tukey’s post hoc test, and presented as mean ± standard deviation (SD). Abbreviations: THB, tetrahydroberberine; PPD, protopanaxadiol; PCPA, p-chlorophenylalanine. Compared with the normal control group: * *p* < 0.05, ** *p* < 0.01, *** *p* < 0.001; compared with the PCPA-induced insomnia model group: ## *p* < 0.01, ### *p* < 0.001; THB and PPD groups vs. combination group: $$ *p* < 0.01.

Sodium pentobarbital-induced sleep tests evaluated rats’ sleep behavior (Figure 1A,B): compared with the p-chlorophenylalanine (PCPA)-induced insomnia model group, the tetrahydroberberine (THB) + protopanaxadiol (PPD) combination group significantly shortened sleep latency (SL) and prolonged sleep duration (SD) (* *p* < 0.01). The THB and PPD monotherapy groups improved these sleep parameters but with weaker effects than the combination group, which also outperformed the positive control (diazepam) group. These results confirm a synergistic effect of THB and PPD in regulating the sleep behavior of insomniac rats. Dynamic monitoring of body weight (Figure 1C) showed that the insomnia model group had significantly slowed weight gain due to chronic sleep disturbance, while all administration groups exhibited improved weight growth. Notably, the THB + PPD combination group had body weight levels closest to the normal control group, indicating that combination therapy effectively alleviates insomnia-induced physiological stress and promotes the recovery of rats’ overall physiological status.

### 2.2. THB Combined with PPD Alleviates Hippocampal Pathological Damage and Regulates Neurochemical Balance

Hematoxylin-eosin (HE) staining of the hippocampus revealed that the dentate gyrus (DG) region of the hippocampus in the p-chlorophenylalanine (PCPA)-induced insomnia model group exhibited typical pathological damage, with disordered cell arrangement. The area indicated by the arrow showed particularly significant damage, with a large number of neurons presenting abnormal states of pyknosis and hyperchromatism. All drug-administered groups alleviated the cellular damage in the hippocampal DG region to varying degrees, and the number of damaged neurons was significantly reduced. Among them, the tetrahydroberberine (THB) combined with protopanaxadiol (PPD) group showed the most intact neuronal morphology, with its arrangement order and cellular structure highly close to the normal level.

A 0–3 grade semi-quantitative scoring standard was formulated to evaluate the pathological damage of the hippocampal DG region, with the criteria defined as follows: Grade 0 represents no damage; Grade 1 represents mild damage; Grade 2 represents moderate damage; and Grade 3 represents severe damage. According to this standard, the normal control group was scored as Grade 0, the PCPA-induced insomnia model group was scored as Grade 3, the THB combined with PPD group was scored as Grade 1, and the THB monotherapy group, PPD monotherapy group, as well as the positive drug group were scored as Grade 2.

**Figure 2:**
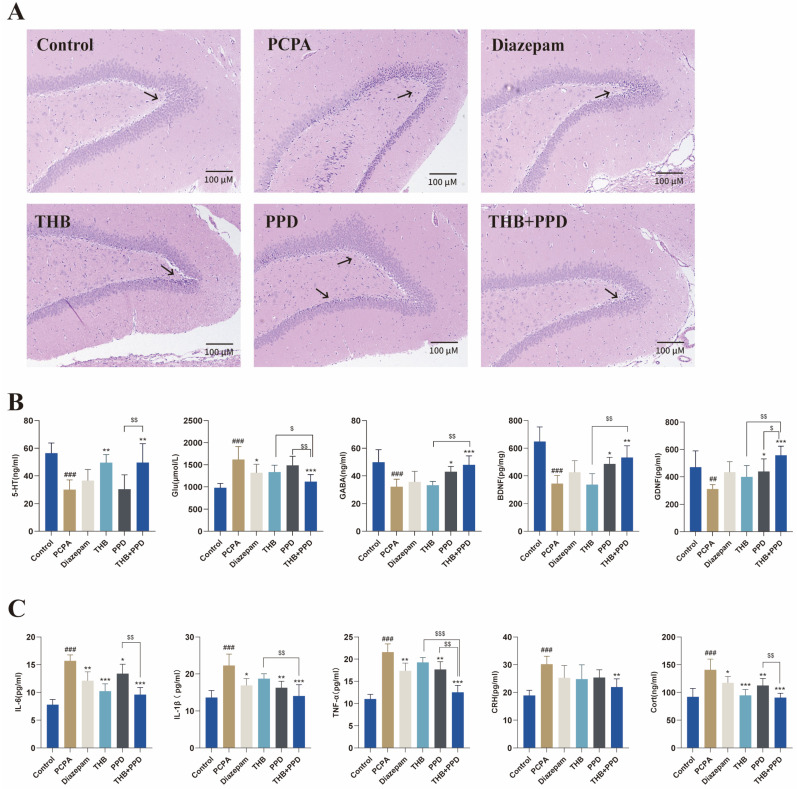
THB + PPD alleviates hippocampal injury and regulates neurochemical balance in insomniac rats: (**A**) HE staining of hippocampal tissue. The area indicated by the arrow is the key observation object, which is the dentate gyrus (DG) region of the hippocampus. (**B**) Levels of neurotransmitters (5-HT, DA, Glu) and neurotrophic factors (BDNF, GDNF) in hippocampus/serum (*n* = 6). (**C**) Levels of HPA axis hormones (CORT, CRH) and pro-inflammatory factors (TNF-α, IL-1β, IL-6) (*n* = 6). Data: one-way ANOVA + Tukey’s test, mean ± SD. vs. normal group: * *p* < 0.05, ** *p* < 0.01,*** *p* < 0.001; vs. PCPA model group: ## *p* < 0.01, ### *p* < 0.001; THB and PPD groups vs. combination group: $ *p* < 0.05, $$ *p* < 0.01, $$$ *p* < 0.001.

These results suggest that the combined medication exerts a clear neuroprotective effect on the hippocampal DG region of insomnia rats (Figure 2A).

Enzyme-linked immunosorbent assay (ELISA) further demonstrated that THB combined with PPD significantly upregulated the levels of sleep–wake regulatory neurotransmitters (5-hydroxytryptamine [5-HT], dopamine [DA], and glutamate [Glu]) in hippocampal tissue or serum, as well as the expression of neurotrophic factors (brain-derived neurotrophic factor [BDNF] and glial cell line-derived neurotrophic factor [GDNF]) (Figure 2B). Additionally, the combination therapy remarkably reduced the levels of hypothalamic–pituitary–adrenal (HPA) axis-related hormones (corticosterone [CORT] and corticotropin-releasing hormone [CRH]) and peripheral/central pro-inflammatory factors (tumor necrosis factor-α [TNF-α], interleukin-1β [IL-1β], and interleukin-6 [IL-6]) (Figure 2C). Collectively, these results indicate that THB combined with PPD can effectively regulate the neurochemical balance of insomniac rats, while mitigating HPA axis overactivation and neuroinflammatory responses associated with sleep disturbance.

### 2.3. THB Combined with PPD Restructures Gut Microbiota Composition and Improves Serum Metabolomic Profile

**Figure 3:**
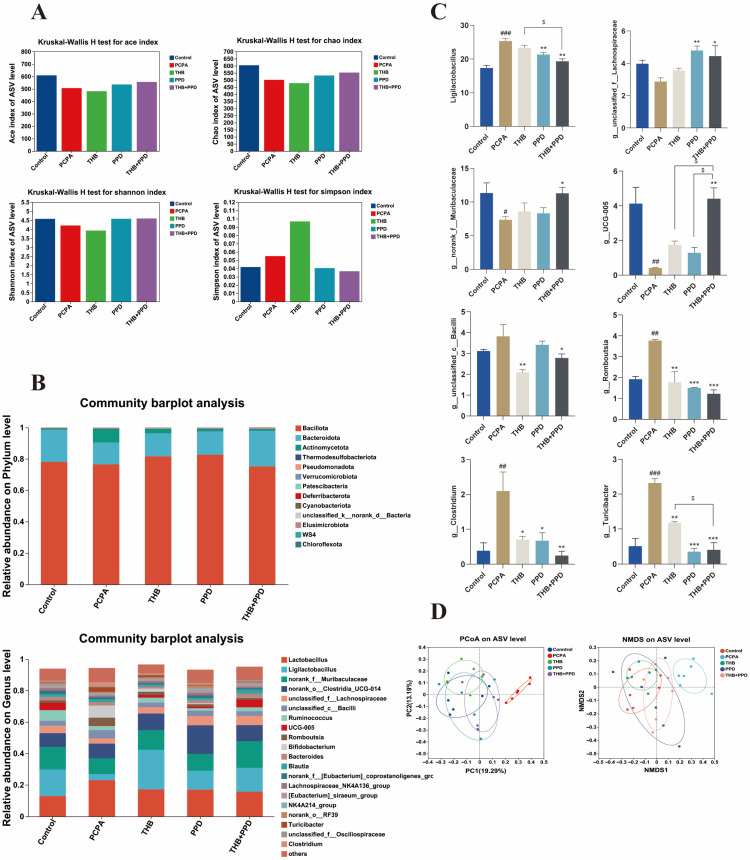
THB + PPD restores gut microbiota structure in insomniac rats: (**A**–**D**) Gut microbiota α/β diversity and relative abundances of beneficial/harmful bacteria (e.g., Lactobacillus) analyzed by 16S rRNA sequencing (*n* = 6). Data: one-way ANOVA + Tukey’s test, mean ± SD. vs. normal group: * *p* < 0.05, ** *p* < 0.01, *** *p* < 0.001; vs. PCPA model group: # *p* < 0.05, ## *p* < 0.01, ### *p* < 0.001; THB and PPD groups vs. combination group: $ *p* < 0.05.

**Figure 4:**
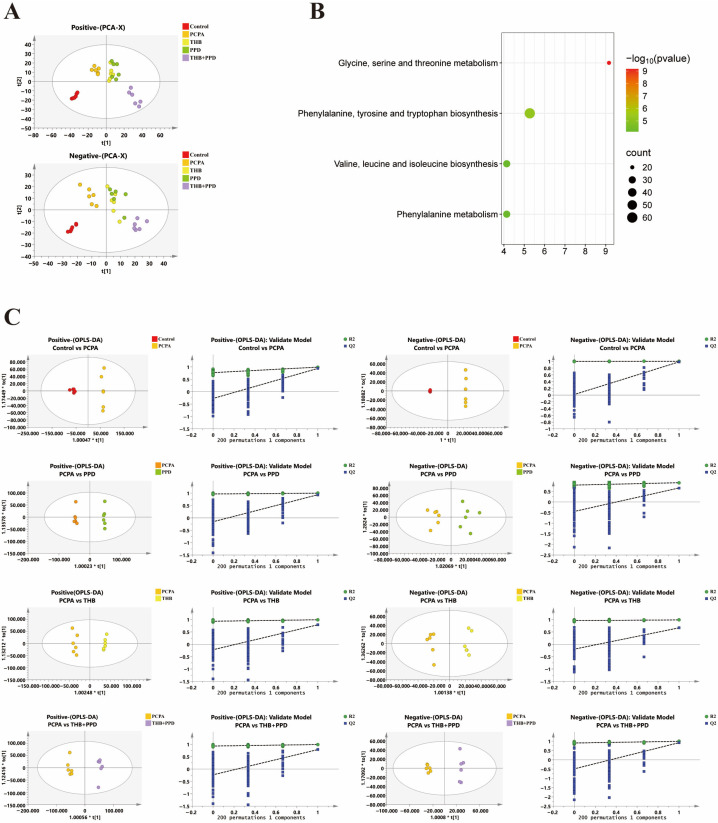
THB + PPD improves serum metabolomic profile in insomniac rats: (**A**–**C**) Serum metabolomic profile clustering and enrichment of key differential metabolites in glycine/serine/threonine metabolism pathways (*n* = 6).

Restoration of gut microecology: 16S rRNA sequencing analysis showed that the combination therapy effectively reversed the PCPA-induced decrease in gut microbiota α- and β-diversity, and significantly adjusted the relative abundances of specific beneficial bacteria (e.g., Lactobacillus) and harmful bacteria back to levels close to those in the normal group (Figure 3A–D). Correction of serum metabolic disorders: Untargeted metabolomic analysis revealed that the combination therapy significantly shifted the serum metabolomic profile of model rats toward that of the normal group. Key differential metabolites were mainly enriched in metabolic pathways such as glycine, serine, and threonine metabolism (Figure 4A–C), suggesting that the restoration of metabolic homeostasis is a crucial link in the therapeutic effect.

### 2.4. The Combined Treatment of THB and PPD Exerts a Synergistic Effect by Regulating Key Signaling Pathways in the Hippocampus

**Figure 5:**
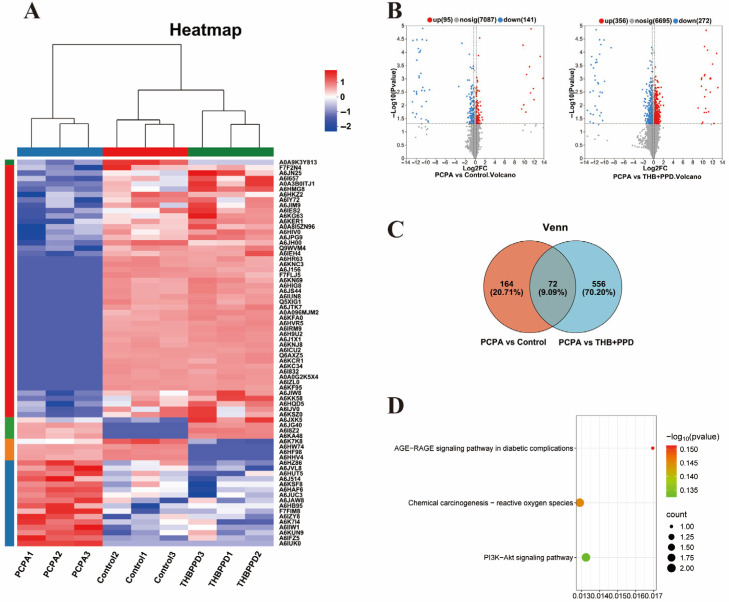
Proteomics analysis identifies core signaling pathways regulated by THB + PPD in hippocampus: (**A**–**D**) Screening and enrichment analysis of PI3K/AKT and AGE-RAGE signaling pathways via hippocampal proteomics (*n* = 6). (**B**) Volcano plot of differentially expressed genes/proteins between PCPA and control groups. The vertical dashed line at Log2FC = 0 separates upregulated (right) and downregulated (left) genes/proteins. The horizontal dashed line at −Log10(*p* value) = 1 indicates the significance threshold (*p* < 0.05). Red dots (*n* = 95) represent significantly upregulated genes/proteins, blue dots (*n* = 141) represent significantly downregulated genes/proteins, and gray dots (*n* = 7087) represent non-significant changes.

**Figure 6:**
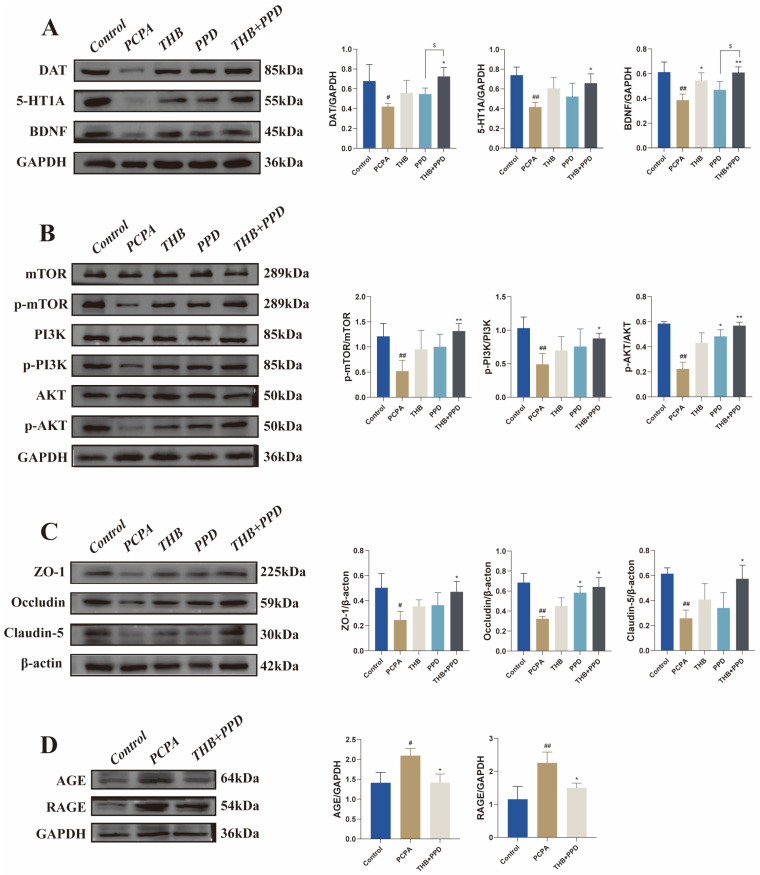
Western blot verification of key proteins and pathways: (**A**) Expression of neurochemical balance-related proteins (5-HT, DAT, BDNF) (*n* = 6). (**B**) Protein expression of PI3K/AKT/mTOR pathway (p-PI3K, p-AKT, p-mTOR) (*n* = 6). (**C**) Expression of tight junction proteins (ZO-1, Occludin) (*n* = 6). (**D**) Protein expression of AGE-RAGE pathway (RAGE) (*n* = 6). Data: one-way ANOVA + Tukey’s post hoc test, mean ± SD. vs. normal group: * *p* < 0.05, ** *p* < 0.01; vs. PCPA model group: # *p* < 0.05, ## *p* < 0.01; THB and PPD groups vs. combination group: $ p < 0.05.

Proteomics reveals core pathways: Hippocampal tissue proteomics analysis identified the PI3K/AKT and AGE-RAGE signaling pathways as the most significantly regulated pathways by the combination therapy (Figure 5A–D). WB verification of key targets: Western blot (WB) results confirmed that the combination therapy significantly activated the PI3K/AKT/mTOR pathway (increasing the protein expression of p-PI3K, p-AKT, and p-mTOR) and significantly inhibited the AGE-RAGE pathway (decreasing the protein expression of RAGE, etc.) (Figure 6B,D). Meanwhile, the expression of tight junction proteins (e.g., ZO-1 and Occludin) was also significantly upregulated (Figure 6C). Mechanism integration: WB also verified the ELISA results, showing changes in the expression of key proteins such as 5-HT, DAT, and BDNF (Figure 6A), which confirmed the improvement of neurochemical balance at the molecular level.

## 3. Discussion

Insomnia, a multifactorial sleep disorder characterized by central nervous system hyperarousal, is closely intertwined with hypothalamic–pituitary–adrenal (HPA) axis dysregulation [18], systemic inflammation, and gut microbiota imbalance. Current therapeutic strategies are limited by side effects, poor accessibility, or single-target actions, highlighting the need for safe and multi-dimensional interventions. The present study systematically explored the therapeutic effects and underlying mechanisms of combined tetrahydroberberine (THB) and protopanaxadiol (PPD) administration on p-chlorophenylalanine (PCPA)-induced insomnia in rats, integrating behavioral, histological, neurochemical, microbiomic, metabolomic, and proteomic analyses. The results not only confirm the synergistic efficacy of this combination but also reveal a comprehensive regulatory network across the microbiota–gut–brain axis (MGBA), providing new insights into insomnia pathophysiology and treatment.

The core phenotypic improvements observed in this study directly validate the synergistic effect of THB and PPD. Combined treatment significantly shortened sleep latency and prolonged sleep duration in insomniac rats, with effects superior to either monotherapy or the diazepam positive control. This sleep-regulating capacity was accompanied by the recovery of body weight, a key indicator of systemic physiological stress alleviation [19]. Chronic insomnia-induced weight gain stems from persistent hyperarousal and metabolic disturbance, and the restoration of weight trajectory in the combined treatment group reflects the holistic therapeutic benefit beyond mere sleep improvement. At the central level, hematoxylin-eosin (HE) staining demonstrated that THB + PPD effectively preserved hippocampal neuron morphology, reversing the disorganization and nuclear pyknosis observed in the model group [20]. The hippocampus, a critical region for sleep regulation and cognitive function, is highly vulnerable to sleep deprivation-induced damage, and the neuroprotective effect of the combination therapy lays a structural foundation for improved neural function.

Neurochemical balance restoration and the suppression of overactive stress and inflammatory responses further underpin the therapeutic mechanism [21]. Enzyme-linked immunosorbent assay (ELISA) and Western blot results collectively showed that THB + PPD upregulated the levels of neurotrophic factors (brain-derived neurotrophic factor [BDNF] and glial cell line-derived neurotrophic factor [GDNF]) and modulated the balance of neurotransmitters (5-hydroxytryptamine [5-HT], dopamine [DA], and glutamate [Glu]). These neurotrophic factors and neurotransmitters form a reciprocal regulatory loop: BDNF and GDNF promote the survival, synaptic plasticity, and functional maintenance of 5-HTergic, DAergic, and glutamatergic neurons, enhancing the synthesis, release, and reuptake of neurotransmitters to correct Glu-induced central overexcitation and restore 5-HT/DA-mediated sleep–wake cycle balance. Conversely, balanced neurotransmitter signaling upregulates the transcription and expression of BDNF/GDNF by activating downstream neuroprotective pathways, reinforcing this homeostatic loop. BDNF and GDNF are essential for neuron survival [22], synaptic plasticity, and neurogenesis, while the precise regulation of monoamine and excitatory neurotransmitters corrects the hyperarousal state characteristic of insomnia [23].

Concurrently, the combination therapy reduced the levels of HPA axis-related hormones (corticosterone [CORT] and corticotropin-releasing hormone [CRH]) [24] and pro-inflammatory factors (tumor necrosis factor-α [TNF-α], interleukin-1β [IL-1β], and interleukin-6 [IL-6]) in both serum and hippocampal tissue. These stress and inflammatory factors are closely interconnected with neurotrophic factors and neurotransmitters: elevated CORT/CRH inhibits BDNF/GDNF expression, impairs neurotransmitter system function, and activates microglia to secrete TNF-α, IL-1β, and IL-6. These pro-inflammatory factors further downregulate 5-HT/DA levels and Glu balance by damaging neurotransmitter receptors, while suppressing BDNF/GDNF synthesis to weaken neuroprotection. In turn, upregulated BDNF/GDNF and restored neurotransmitter balance inhibit HPA axis hyperactivity and microglia activation, reducing CORT/CRH and pro-inflammatory factor release, forming a protective regulatory network [25]. HPA axis hyperactivity is a well-established driver of insomnia, as elevated glucocorticoid levels enhance central arousal signals [26]; meanwhile, peripheral and central inflammation creates a pro-arousal microenvironment by disrupting neural signaling. The dual regulation of these two interconnected systems—stress response and inflammation—addresses a key pathological hub of insomnia [27].

A critical finding of this study is the role of gut microbiota remodeling in mediating the therapeutic effects of THB + PPD, highlighting the MGBA as a central regulatory pathway [28]. Notably, common insomnia medications often exert adverse effects on gut microbiota homeostasis: benzodiazepines and non-benzodiazepine hypnotics reduce the abundance of beneficial bacteria (e.g., Bifidobacterium and Lactobacillus) and impair short-chain fatty acid synthesis; melatonin agonists disrupt the circadian synchronization of gut microbiota; and antidepressant hypnotics alter intestinal neurotransmitter metabolism, leading to dysbiosis and low-grade inflammation, which may weaken long-term therapeutic efficacy via MGBA dysfunction [29].

In contrast, 16S rRNA sequencing revealed that combined treatment reversed PCPA-induced reductions in gut microbiota α- and β-diversity and restored the relative abundance of beneficial bacteria to levels comparable to the normal group [30]. This microbial restructuring was tightly linked to metabolic homeostasis, as untargeted metabolomics identified glycine–serine–threonine metabolism as a key regulated pathway [31]. Glycine, a major inhibitory neurotransmitter in the central nervous system, exerts direct sedative effects by enhancing postsynaptic inhibition [32], while threonine modulates sleep drive through interactions with GABAergic signaling [33]. These metabolites serve as molecular messengers, transmitting peripheral microbial signals to the central nervous system via the circulatory system [34]. The correlation between core microbiota abundance, key metabolites, and sleep parameters further supports a causal link in the “microbiota–metabolite–CNS” regulatory axis, suggesting that microbial remodeling is an upstream initiating event of the combined therapy’s efficacy.

The synergistic regulation of key signaling pathways in the hippocampus integrates peripheral and central mechanisms, forming a comprehensive therapeutic network. Proteomic and Western blot analyses identified the PI3K/AKT/mTOR and AGE-RAGE pathways as core targets of THB + PPD. Activation of the PI3K/AKT/mTOR pathway promotes BDNF expression, enhances neuron survival, and improves synaptic plasticity—effects that directly contribute to hippocampal protection and neurotransmitter balance restoration [35]. In parallel, inhibition of the AGE-RAGE pathway reduces pro-inflammatory signaling, as AGE-RAGE activation is a potent amplifier of NF-κB-mediated cytokine production [36]. The crosstalk between these pathways creates a “neuroprotection-anti-inflammation” synergy: reduced inflammation alleviates the pathological microenvironment [37], while enhanced neurotrophic signaling promotes neural repair. Additionally, the upregulation of tight junction proteins (ZO-1 and Occludin) suggests improved blood–brain barrier integrity, which further protects central nervous system homeostasis by limiting the entry of peripheral inflammatory factors [38].

Integrating these findings, we propose a unifying mechanism for the synergistic effect of THB and PPD: combined administration first reshapes gut microbiota composition, increasing the abundance of beneficial bacteria and regulating glycine–serine–threonine metabolism. These peripheral changes are transmitted to the central nervous system via the MGBA, inhibiting HPA axis hyperactivity and AGE-RAGE-mediated neuroinflammation while activating the PI3K/AKT/mTOR neurotrophic pathway. Ultimately, these multi-target actions converge to restore neurochemical balance, protect hippocampal structure, and improve sleep architecture. This network-based regulation aligns with the complex pathophysiology of insomnia and explains the superior efficacy of combined therapy compared to single-agent interventions.

This study has several limitations that should be acknowledged. The sample size (*n* = 6 per group) is relatively small, which may affect the statistical power and generalizability of the results. While multi-omics correlation analyses strongly suggest a causal link within the MGBA, direct evidence from fecal microbiota transplantation experiments is lacking to confirm the independent role of gut microbiota [39]. Additionally, the optimal dosage ratio, administration duration, and pharmacokinetic interactions of THB and PPD remain to be fully elucidated, which are critical for translational application. Future studies should expand the sample size, conduct fecal microbiota transplantation to verify microbial causality, optimize the drug combination ratio, and explore pharmacokinetic synergies. Clinical trials are also warranted to validate the therapeutic potential of THB + PPD in human insomnia patients.

In summary, this study demonstrates that combined THB and PPD exerts synergistic therapeutic effects on PCPA-induced insomnia in rats through a multi-layered mechanism involving the MGBA and key signaling pathways. The combination therapy effectively integrates peripheral microbial-metabolic regulation with central neuroprotective and anti-inflammatory actions, addressing the complex pathophysiology of insomnia. These findings not only deepen our understanding of MGBA-mediated sleep regulation but also provide a novel, safe, and multi-target strategy for insomnia treatment. The synergistic efficacy of THB and PPD highlights the value of natural product combinations in developing novel therapeutics for neuropsychiatric disorders, laying a solid foundation for future translational research.

## 4. Experimental Procedures

### 4.1. Chemicals and Reagents

Tetrahydroberberine (THB, purity >99%) and protopanaxadiol (PPD, purity >99%) were supplied by Shandong Xinshidai Pharmaceutical Co., Ltd. (Linyi, China). Para-chlorophenylalanine (PCPA, MW: 199.6 Da) was obtained from Sigma-Aldrich (St. Louis, MO, USA).

### 4.2. Insomnia Rat Model and Administration

Thirty-six SPF-grade male Sprague-Dawley (SD) rats (6–8 weeks old, 200 ± 20 g) were purchased from Pengyue Biotechnology Co., Ltd. (Jinan, China; animal production license: SCXK (Lu) 2022-0006). The study was approved by the Laboratory Animal Care and Use Committee of Lunan Pharmaceutical Group Co., Ltd., Linyi China (approval No.: AN-IACUC2024-118) and conducted in strict accordance with the Guide for the Care and Use of Laboratory Animals to ensure animal welfare. Rats were randomized into 6 groups by body weight (*n* = 6/group): control (no modeling, solvent only), PCPA (insomnia model, modeling + solvent), diazepam (positive control, modeling + diazepam 0.2 mg/kg), THB (modeling + tetrahydroberberine 6.25 mg/kg), PPD (modeling + protopanaxadiol 45 mg/kg), and THB + PPD (modeling + tetrahydroberberine 6.25 mg/kg + protopanaxadiol 45 mg/kg). For drug preparation: Diazepam, THB, and PPD were dissolved in purified water to desired concentrations; p-chlorophenylalanine (PCPA) solution (30 mg/mL) was prepared with dimethyl sulfoxide (DMSO), Tween-80, and normal saline as mixed solvent. Insomnia model establishment: Control rats were given equal-volume PCPA solvent (1% DMSO, 0.5% Tween-80, and normal saline as balance) via intraperitoneal injection once daily for 3 days. PCPA group and administration groups received intraperitoneal injection of PCPA solution (300 mg/kg) once daily for 3 days, with the same carrier ratio applied to maintain uniform vehicle conditions among all groups [40]. Model success was confirmed by behavioral signs: hyperarousal (sensitivity to mild stimuli and startle reflex), increased aggressiveness (more cage-mate fights), and elevated spontaneous activity. After successful modeling, administration groups received corresponding drugs by gavage (10 mL/kg), while control and PCPA groups received equal-volume purified water, once daily for 7 days. Rats’ general status (body weight, diet, and behavior) was monitored throughout.

### 4.3. Pharmacodynamic Studies and Sample Collection

During the experiment, researchers monitored rats’ daily behavioral status (spontaneous activity, alertness, and aggressiveness) and physiological indicators (food intake and water intake), and weighed them weekly at fixed times to record body weight trends. One hour after the first administration, a sodium pentobarbital-induced sleep test was performed to assess sleep quality: sodium pentobarbital was intraperitoneally injected at 40 mg/kg based on body weight, with injection completion as the timing start. Sleep latency (SL, time from injection end to loss of righting reflex) and sleep duration (SD, time from righting reflex loss to recovery) were recorded to evaluate the drugs’ effects on shortening SL and prolonging SD in insomniac rats [41]. After 7 days of administration, rats were anesthetized via intramuscular injection of a mixed anesthetic (zolazepam 20 mg/kg + xylazine hydrochloride 10 mg/kg). Once the corneal reflex disappeared and muscles relaxed, abdominal aortic venous blood was collected, centrifuged at 3500 r/min at 4 °C for 10 min to separate serum supernatant, which was aliquoted and stored at −80 °C for subsequent detection of inflammatory factors, neurotransmitters and other indicators. After blood collection, rats were sacrificed by decapitation; the whole brain was divided into two parts: one for Western blot analysis (e.g., neurotrophic factors and signaling pathway proteins) with relevant kits, and the other fixed in 4% paraformaldehyde solution (5–10 times the tissue volume) at 4 °C for 24 h for subsequent hematoxylin-eosin (HE) staining to assess brain tissue pathological damage. Additionally, 24 h before sacrifice, fresh fecal samples were collected into enzyme-free cryopreservation tubes (labeled with group and number) and immediately stored at −80 °C for subsequent intestinal flora analysis.

### 4.4. Hematoxylin-Eosin Staining (H&E)

The brain tissues were fixed in 4% paraformaldehyde solution for 48 h, routinely embedded in paraffin, and coronally sectioned to include the hippocampal region. Hematoxylin-eosin (HE) staining was performed using standard staining solutions. Morphological and pathological changes in the dentate gyrus (DG) area of the hippocampus were observed under a microscope. Based on the HE staining results, neuronal damage in the hippocampal region caused by the insomnia model was analyzed by evaluating neuronal arrangement density, nuclear pyknosis, hyperchromasia, and somatic swelling/shrinkage [42].

### 4.5. Enzyme-Linked Immunosorbent Assay (ELISA)

According to the manufacturer’s instructions of the ELISA kits, the contents of brain-derived neurotrophic factor (BDNF) and glial cell line-derived neurotrophic factor (GDNF) in hippocampal tissue extracts of rats were determined, as well as the levels of tumor necrosis factor (TNF)-α, interleukin (IL)-1β, IL-6, corticosterone (Cort), corticotropin-releasing hormone (CRH), γ-aminobutyric acid (GABA), glutamate (Glu), 5-hydroxytryptamine (5-HT), and dopamine (DA) in rat serum.

### 4.6. Western Blot Analysis

Western blot analysis was performed to detect protein expression in brain tissue. Proteins were extracted from the brain tissues, and their concentrations were determined according to the instructions of the BCA kit. A total of 40 μg of protein per sample was separated by 10% SDS-PAGE (sodium dodecyl sulfate–polyacrylamide gel electrophoresis) and transferred onto a PVDF membrane. The membrane was blocked with 5% skim milk for 2 h at room temperature. Subsequently, it was incubated with the primary antibody at 4 °C overnight, followed by incubation with the corresponding HRP-conjugated secondary antibody for 2 h at room temperature. Protein bands were visualized using the ChemiScope 6200 automated chemiluminescence imaging system, and the gray values were analyzed with ImageJ software (ImageJ 1.51j8).

Primary Antibodies: DAT (AB184451); 5-HT1A (AB856150); BDNF (AB108319); Rabbit GAPDH (AF2819); mTOR (2983S); p-mTOR (5536S); PI3K (AB191606); p-PI3K (17366S); Akt (9272S); p-Akt (4060S); ZO-1 (AB276131); Occludin (AB216327); Claudin-5 (13255S); β-acton (AF2811); AGE (AB23722); RAGE (AB216329).

Secondary Antibodies: Horseradish peroxidase (HRP)-conjugated goat anti-mouse IgG (A0216); HRP-conjugated goat anti-rabbit IgG (A0208).

### 4.7. Gut Microbiota Analysis

On the day before the end of the experiment, fresh fecal samples were collected from rats under aseptic conditions, placed in sterile cryotubes, and stored at −80 °C. Total genomic DNA was extracted from the fecal samples using the E.Z.N.A.^®^ Stool DNA Kit (Omega Bio-tek, Inc., Norcross, GA, USA) according to the manufacturer’s instructions. The V3–V4 hypervariable region of the 16S rRNA gene was amplified using the primers 338F (5′-ACTCCTACGGGAGGCAGCAG-3′) and 806R (5′-GGACTACHVGGGTWTCTAAT-3′). The PCR products were quantified and normalized using the QuantiFluor™-ST Blue Fluorescence Quantification System (Promega). After quality assessment, Illumina sequencing was performed: paired-end (PE) reads obtained from Illumina sequencing were demultiplexed into samples. Quality control and filtering of the raw PE reads were conducted based on sequencing quality, followed by merging of paired-end reads using overlap relationships to generate high-quality clean tags. The clean data were then processed with noise reduction algorithms (e.g., DADA2 or Deblur) to obtain amplicon sequence variants (ASVs) and abundance data. Based on the representative sequences and abundance information of ASVs, microbiome analysis and visualization were performed using the Majorbio Cloud Platform (https://cloud.majorbio.com, 15 April 2025).

### 4.8. Non-Targeted Metabolomics Techniques

The procedures for serum metabolomics analysis were performed as follows: 110 μL of serum sample was added to 220 μL of a methanol and acetonitrile mixture (1:1, *v*/*v*). The mixture was thoroughly vortexed and sonicated for 10 min, and this step was repeated twice. The sample was then centrifuged at 11,000 r for 15 min at 4 °C to obtain the supernatant. After centrifugation, the supernatant was transferred into an injection vial for instrumental analysis.

Chromatographic separation was carried out using a Vanquish ultra-performance liquid chromatography (UPLC) system equipped with a Waters ACQUITY UPLC HSS T3 column (2.1 mm × 100 mm, 1.8 μm). The separated compounds were subsequently analyzed by mass spectrometry using an LTQ Orbitrap tandem mass spectrometer.

For data processing, peak extraction was performed using Xcalibur software (Xcalibur 4.3), and chromatographic peaks were quantified with Compound Discover 3.2 software. Multivariate statistical analyses, including principal component analysis (PCA) and orthogonal partial least squares-discriminant analysis (OPLS-DA), were conducted using SIMCA-14.1 software.

### 4.9. Proteomics Research

All experimental procedures in the proteomics study were performed according to the methods described by Sun et al. Data analysis was carried out using the free online platform available on the Majorbio Cloud (https://cloud.majorbio.com, accessed on 27 April 2025). Significant differences between groups were assessed using the *t*-test function in R to calculate p-values and fold change (FC). A significance threshold of *p* < 0.05 and a fold change greater than 2 were applied to identify differentially expressed proteins. Metabolic pathway analysis was conducted using the KEGG (Kyoto Encyclopedia of Genes and Genomes, https://www.genome.jp/kegg/, accessed on 29 April 2025) pathway database.

### 4.10. Statistical Analysis

All statistical analyses were performed using GraphPad Prism software (Version 8.0, GraphPad Software, Inc., San Diego, CA, USA). Prior to statistical testing, the Shapiro–Wilk test was used to verify the normality of data distribution. For comparisons between two groups, unpaired Student’s *t*-test was applied. For multiple group comparisons, one-way analysis of variance (ANOVA) was conducted, followed by Tukey’s post hoc test to correct for multiple comparisons and ensure the reliability of pairwise comparisons. Spearman’s rank correlation analysis was performed to assess the relationships between gut microbial taxa, serum metabolites, and sleep parameters (sleep latency and sleep duration). A sample size of 6 rats per group was determined based on previous similar studies and power analysis (power = 0.8, α = 0.05) to ensure sufficient statistical power. All data are presented as mean ± standard deviation (SD), and a two-tailed *p*-value < 0.05 was considered statistically significant.

## 5. Conclusions

Collectively, our findings confirm that tetrahydroberberine combined with protopanaxadiol exerts synergistic therapeutic effects on PCPA-induced insomnia in rats. The underlying mechanism involves bidirectional regulation of the microbiota–gut–brain axis, activation of the PI3K/AKT neurotrophic pathway, and inhibition of the AGE-RAGE pro-inflammatory pathway, which jointly restore neurochemical balance, alleviate neuroinflammation, and improve sleep architecture. This study provides a novel multi-target strategy for clinical insomnia treatment and lays a foundation for the development of safe and effective combined natural product-based therapeutics.

## Data Availability

The original contributions presented in this study are included in the article. Further inquiries can be directed to the corresponding authors.
